# Pathogenic, glycolytic PD-1^+^ B cells accumulate in the hypoxic RA joint

**DOI:** 10.1172/jci.insight.139032

**Published:** 2020-11-05

**Authors:** Achilleas Floudas, Nuno Neto, Viviana Marzaioli, Kieran Murray, Barry Moran, Michael G. Monaghan, Candice Low, Ronan H. Mullan, Navin Rao, Vinod Krishna, Sunil Nagpal, Douglas J. Veale, Ursula Fearon

**Affiliations:** 1 Molecular Rheumatology, Trinity Biomedical Sciences Institute,; 2 Department of Mechanical and Manufacturing Engineering, and; 3 Trinity Centre for Biomedical Engineering, Trinity College Dublin, Dublin, Ireland.; 4 EULAR Centre of Excellence, Centre for Arthritis and Rheumatic Diseases, St. Vincent’s University Hospital, University College Dublin, Dublin, Ireland.; 5 School of Biochemistry and Immunology, Trinity Biomedical Sciences Institute, Trinity College Dublin, Dublin, Ireland.; 6 Department of Rheumatology, Tallaght University Hospital, Dublin, Ireland.; 7 Janssen Research & Development, Immunology, Spring House, Pennsylvania, USA.

**Keywords:** Cell Biology, Metabolism, B cells, Rheumatology, hypoxia

## Abstract

While autoantibodies are used in the diagnosis of rheumatoid arthritis (RA), the function of B cells in the inflamed joint remains elusive. Extensive flow cytometric characterization and SPICE algorithm analyses of single-cell synovial tissue from patients with RA revealed the accumulation of switched and double-negative memory programmed death-1 receptor–expressing (PD-1–expressing) B cells at the site of inflammation. Accumulation of memory B cells was mediated by CXCR3, evident by the observed increase in CXCR3-expressing synovial B cells compared with the periphery, differential regulation by key synovial cytokines, and restricted B cell invasion demonstrated in response to CXCR3 blockade. Notably, under 3% O_2_ hypoxic conditions that mimic the joint microenvironment, RA B cells maintained marked expression of MMP-9, TNF, and IL-6, with PD-1^+^ B cells demonstrating higher expression of CXCR3, CD80, CD86, IL-1β, and GM-CSF than their PD-1^–^ counterparts. Finally, following functional analysis and flow cell sorting of RA PD-1^+^ versus PD-1^–^ B cells, we demonstrate, using RNA-Seq and emerging fluorescence lifetime imaging microscopy of cellular NAD, a significant shift in metabolism of RA PD-1^+^ B cells toward glycolysis, associated with an increased transcriptional signature of key cytokines and chemokines that are strongly implicated in RA pathogenesis. Our data support the targeting of pathogenic PD-1^+^ B cells in RA as a focused, novel therapeutic option.

## Abstract 2

A pathogenic glycolytic B cell population at the site of inflammation in patients with Rheumatoid Arthritis associates with disease severity.

## Graphical abstract

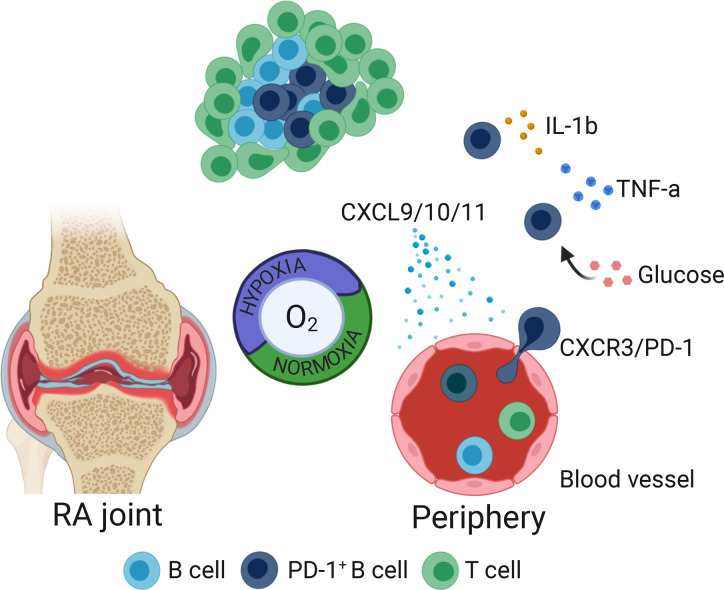

## Introduction

Rheumatoid arthritis (RA) is the most common inflammatory arthropathy and is characterized primarily by the presence of circulating autoantibodies, first described in the 1940s. It often has a progressive and debilitating course, with significant impact on the patient’s quality of life. Despite the long-known association with autoantibodies, knowledge of the role of B cells and their potential direct contribution to disease pathogenesis in RA is limited (1). The recent observation of increased expression of programmed death-1 receptor (PD-1) in subjects who are autoantibody positive, even before they develop RA, suggests this is a primary immune dysregulation in the disease (2). Until recently, the main focus of PD-1 expression and therapeutic targeting of this pathway has been on T cells in the immune response of patients with cancer, which interestingly has led some patients to develop autoimmune diseases, including arthritis (3).

Synovial accumulation of B cells correlates with increased radiographic scores and T cell activation in patients with RA; consequently, B cell–targeting therapies have demonstrated promising results for the treatment of RA, with rituximab (anti-CD20) showing significant efficacy and amelioration of disease progression in patients naive to methotrexate and those with incomplete responses to a TNF inhibitor (4–7). B cell depletion leads to significant but limited reduction in anti-citrullinated protein antibodies (ACPAs) and rheumatoid factor (RF), with studies showing clinical benefit for both autoantibody-positive and autoantibody-negative patients with RA (8, 9). While the exact mechanism leading to disease amelioration following B cell depletion is not fully elucidated, at-risk individuals who received a single dose of rituximab had a significant delay in disease onset that did not correlate with a reduction in IgG-RF or ACPA (10, 11). These studies highlight a key role for B cells at an early stage of disease pathogenesis in RA, in addition to their capacity to produce potentially autoreactive antibodies.

Several aspects of B cell depletion remain poorly understood, which if uncovered, could exert a significant influence on therapeutic outcomes. Recent studies show a population of CD20^+^ T cells is subject to rituximab-mediated depletion (12). These cells express high levels of proinflammatory cytokines IL-17, TNF-α, and IFN-γ, and their potential contribution to the therapeutic effect of rituximab needs to be taken into consideration (13). Plasmablasts and plasma cells do not express CD20 and therefore are not affected by rituximab-mediated B cell depletion. A potential exception, however, has been described in a mouse model of inflammatory arthritis, where short-lived plasma cells residing in the spleen and secondary lymph nodes were shown to express CD20 and thus subject to depletion (14). These cells were preferentially autoreactive, highlighting that although overall antibody titers might remain unchanged following B cell depletion, certain antibody antigen specificities might be preferentially lost. Another complication that arises with current B cell–depleting strategies is the composition and immunological effect of B cell repopulation and the influence that may have on subsequent immune responses. Studies on B cell repopulation, following B cell depletion, have shown that returning B cells are primarily naive, immature, and enriched for IL-10–expressing cells (15, 16).

Therefore, we hypothesized that there are missed opportunities for targeted therapeutic intervention that could potentially minimize off-target effects of B cell depletion by focusing either on the migration of memory B cells to the inflamed RA joint or on specific pathogenic B cell subpopulations, leaving the majority of the B cell pool intact. We have therefore performed extensive characterization of B cell subpopulations in the peripheral blood, synovial fluid, and synovial tissue of patients with early RA and have identified CXCR3 as a major contributor of memory B cell migration to the synovial tissue in RA. Importantly, a subpopulation of CXCR3^+^ B cells constitutively expresses PD-1. PD-1^+^ RA B cells maintained higher T cell costimulatory capacity and proinflammatory cytokine production than their PD-1^–^ counterparts, under normoxic and hypoxic conditions that more closely resembled the environment of the inflamed RA joint. PD-1^+^ B cells accumulated at the site of inflammation in RA, showed increased activation of key metabolic pathways, including AKT/mTOR/S6 signaling pathway activity; and were dependent on STAT3 activation and glucose uptake. Using emerging noninvasive fluorescent lifetime imaging microscopy (FLIM) metabolic imaging technique and RNA-Seq, we show that these RA PD-1^+^ B cells are hyperactive and demonstrate increased glycolytic capacity.

## Results

### Accumulation of double-negative and switched memory B cells at the synovial tissue of RA patients.

**Figure 1:**
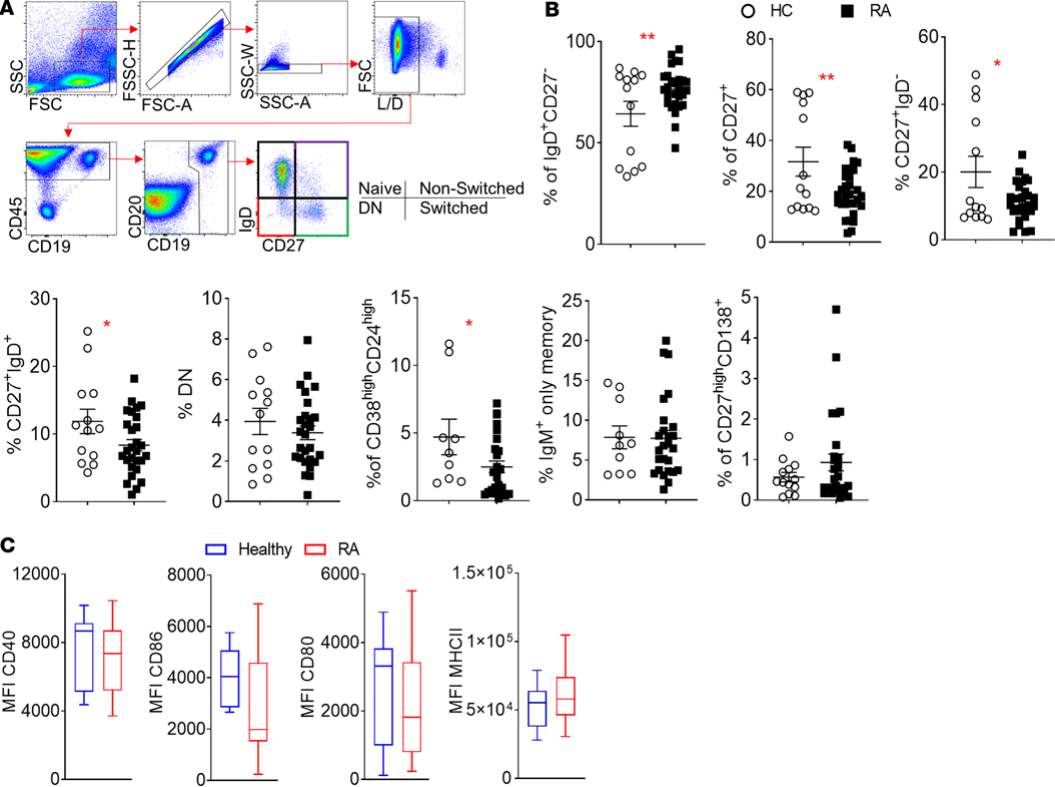
RA patient peripheral blood B cell subpopulation distribution. (**A**) Representative flow cytometric analysis gating strategy for the identification of naive (IgD^+^CD27^–^), non-switched memory (IgD^+^CD27^+^), switched memory (IgD^–^CD27^+^), and double-negative memory (IgD^–^CD27^–^) B cells (over 20 independent experiments performed). DN, double-negative; L/D, LIVE/DEAD stain. (**B**) Frequency of the indicated B cell populations in the PBMCs of HC (*n* = 9–13) and RA patients (*n* = 18–30). Data are presented as mean ± SEM. Each symbol represents an individual sample. Statistical analysis was performed by using standard Student’s *t* test. *P < 0.05. (**C**) MFI values for the expression of CD40, CD86, CD80, and MHCII (HLA-DR) for HC and RA patient peripheral blood CD19^+^CD20^+^ B cells. Data are represented as a box-and-whisker plot, with bounds from 25th to 75th percentile, median line, and whiskers ranging from 5th to 95th percentile. Data are presented as mean ± SEM; each symbol represents an individual sample. Statistical analysis was performed by using 1-way ANOVA with Tukey’s multiple-comparisons test. *P < 0.05, **P < 0.01.

Multiparametric flow cytometric analysis for the identification of naive (IgD^+^CD27^–^), CD27^+^ memory, non–switched memory (IgD^+^CD27^+^), switched memory (IgD^–^CD27^+^), double-negative memory (IgD^–^CD27^–^), transitional (CD24^hi^CD38^hi^), and IgM-only memory (IgD^–^CD27^+^IgM^+^) B cell subpopulations and plasma cells (CD138^+^CD27^hi^) was performed (Figure 1A). Unexpectedly, a highly significant (*P* = 0.0022) increased frequency of naive B cells in the peripheral blood of healthy controls (HCs) compared with patients with RA was observed (Figure 1B). This difference was coupled with a significantly (*P* = 0.009) reduced frequency of CD27^+^ memory B cells in RA compared with HC that was similarly distributed between switched (*P* = 0.02) and non–switched memory (*P* = 0.04) B cells (Figure 1B). Although no significant differences were observed in the frequency of IgM-only memory B cells or plasma cells between RA and HC, a significant reduction in the frequency of transitional CD24^hi^CD38^hi^ enriched for IL-10–producing B cells was observed in RA patient compared with HC peripheral blood (*P* = 0.04) (Figure 1B). Analysis of T cell costimulatory molecules of RA patient and HC peripheral blood B cells revealed no significant differences in the expression of CD80, CD86, HLA-DR, or CD40 (Figure 1C).

**Figure 2:**
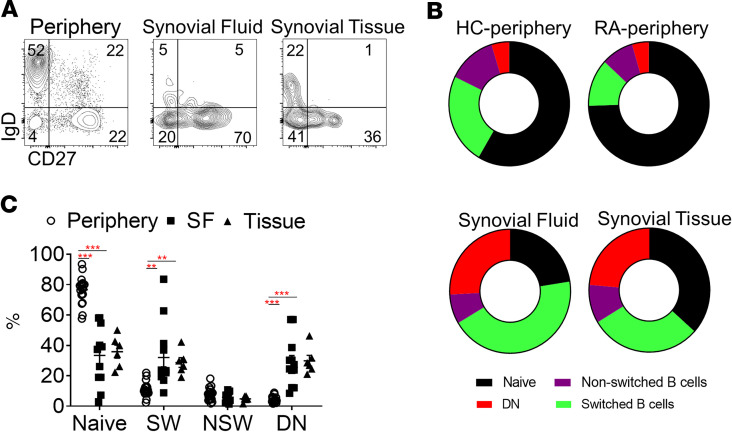
Synovial tissue accumulation of DN and switched memory B cells in RA. (**A**) Representative plots of CD19^+^CD20^+^ B cells for the expression of IgD and CD27 in the peripheral blood, SF, and synovial tissue (at least 5 independent experiments performed). (**B**) Average subpopulation distribution of peripheral blood HC and RA patient B cells and RA patient SF and synovial tissue B cells. (**C**) Frequency of the indicated B cell populations in the periphery (*n* = 20), SF (*n* = 12), and synovial tissue (*n* = 6) of RA patients. Data are presented as mean ± SEM. Each symbol represents an individual sample. Statistical analysis was performed by using 1-way ANOVA with Tukey’s multiple-comparisons test. **P < 0.01, ***P < 0.001.

We then performed an extensive characterization of B cell subpopulations in RA patient synovial fluid (SF) and enzymatically and mechanically digested synovial tissue. While the frequency of CD19^+^CD20^+/–^ cells was significantly lower in RA patient SF (*P* = 0.0005) and synovial tissue (*P* = 0.02) compared with peripheral blood, the subpopulation distribution was markedly different (Supplemental Figure 1 and Supplemental Figure 2; supplemental material available online with this article; https://doi.org/10.1172/jci.insight.139032DS1). Interestingly, a significant reduction in naive (IgD^+^CD27^–^) B cells and a dramatic increase in the frequency of switched memory and double-negative memory B cells in RA SF (*P* < 0.0001 for all) and synovial tissue (*P* < 0.0001, *P* = 0.01, *P* = 0.0002, respectively) compared with peripheral blood was observed (Figure 2, A–C).

### CXCR3 is an important mediator for the accumulation of memory B cells to the site of inflammation in RA.

**Figure 3:**
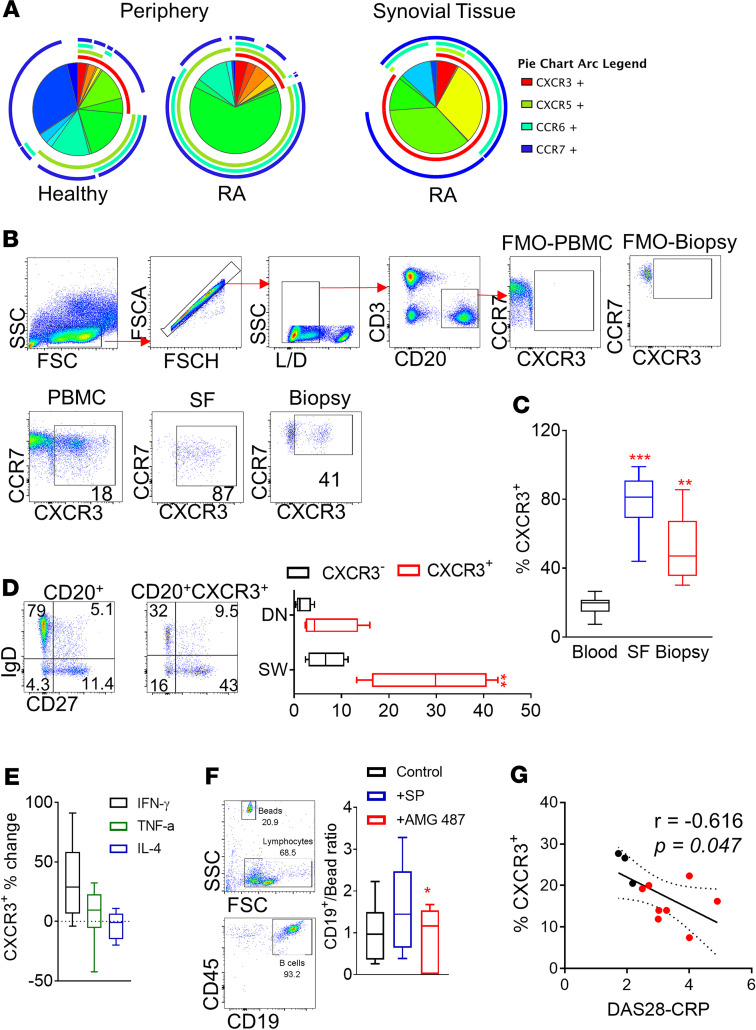
Involvement of CXCR3 in the migration of peripheral blood memory B cells to the synovial tissue. (**A**) SPICE algorithm flow cytometric analysis of peripheral blood and synovial tissue RA patient B cell expression of the chemokine receptors CXCR3, CXCR5, CCR6, and CCR7. (**B**) Representative gating followed for the flow cytometric analysis and identification of CXCR3-expressing peripheral blood, SF, and synovial tissue B cells (at least 6 independent experiments were performed). FMO, fluorescence minus one. (**C**) Frequency of RA patient CXCR3-expressing B cells in the periphery (*n* = 12), SF (*n* = 7), and synovial tissue (*n* = 6). Data are presented as mean ± SEM. Statistical analysis was performed by using 1-way ANOVA with Tukey’s multiple-comparisons test. **P < 0.01, ***P < 0.001. (**D**) Representative flow cytometric analysis plots and frequency of RA patient switched memory and DN memory B cells within the CXCR3^+^ peripheral blood B cell compartment (*n* = 4). Data are presented as mean ± SEM. Ordinary 2-way ANOVA with Holm-Šidák multiple-comparisons test was performed. (**E**) Effect of CXCR3 expression change following incubation of isolated RA patient–derived peripheral blood B cells with the indicated cytokines (*n* = 6/group). (**F**) Representative flow cytometric analysis and CD19^+^/counting bead ratio for of invading B cells toward cRPMI (control), RA synovial biopsy-conditioned media (SP), or RA synovial biopsy-conditioned media following treatment of the B cells with the CXCR3 small molecule antagonist AMG487 (*n* = 7/group, 3 independent experiments); 1-way ANOVA with Tukey’s multiple-comparisons test; *P < 0.05. Data are represented as a box-and-whisker plot, with bounds from 25th to 75th percentile, median line, and whiskers ranging from 5th to 95th percentile. (**G**) Linear regression analysis between the frequency of RA patient peripheral blood CXCR3^+^ B cells and DAS28; *n* = 11; each symbol represents an individual sample. Spearman *r* correlation analysis was performed.

We then examined the potential chemokine receptors involved in the accumulation of switched memory and DN memory B cells at the inflamed joint in RA. An extensive characterization of chemokine receptors was performed by flow cytometric analysis of the peripheral blood, SF, and synovial tissue B cells, including CXCR3, CXCR5, CCR6, and CCR7 (Figure 3, A and B). Chemokine receptor expression pattern differences were observed between HC and RA patient–derived peripheral blood. Such differences could potentially be utilized as novel diagnostic tools (Figure 3A). A significant increase in the expression of the chemokine receptor CXCR3 was evident for RA SF (*P* < 0.0001) and synovial tissue (*P* = 0.0007) B cells compared with peripheral blood B cells (Figure 3, B and C). A relatively small population (approximately 20%) of peripheral blood B cells expressed CXCR3, in comparison with approximately 80% positivity observed at the site of inflammation (Figure 3C). Peripheral blood CXCR3^+^ B cells belonged primarily to the switched memory and DN memory B cell subpopulations, therefore closely resembling the frequency of these cells at the RA synovial tissue (Figure 3, D and F), suggesting that CXCR3 is an important mediator of memory B cell accumulation from the periphery to the site of inflammation in RA. The local microenvironment of the inflamed joint could further contribute to B cell CXCR3 expression since CXCR3 is inducible by TNF and IFN-γ, 2 common proinflammatory cytokines of the joint microenvironment, but suppressed by IL-4 (Figure 3E). RA patient–derived B cells had the capacity to invade in response to RA synovial biopsy-conditioned media; however, upon treatment with the CXCR3 antagonist AMG487, B cell invasion was significantly reduced (*P* = 0.03) (Figure 3F). Importantly, there was a significant inverse correlation (*r* = –0.6, *P* = 0.047) between the peripheral blood frequency of CXCR3^+^ B cells and DAS28-CRP in patients with RA, potentially due to increased migration of CXCR3-expressing B cells to the site of inflammation in patients with higher disease severity, therefore further highlighting the importance of CXCR3 expression for the migration of B cells to the inflamed joint and disease progression (Figure 3G). Interestingly, 6 months to 1 year following rituximab-mediated B cell depletion, the returning B cells were primarily transitional B cells (*P* < 0.0001) expressing high levels of CD5 (associated with regulatory B cell function) (*P* = 0.0042) and CXCR3 (*P* < 0.0001) compared with B cells of patients who had not received B cell depletion therapy (Supplemental Figure 3). These studies highlight an important role for CXCR3 in the accumulation of memory B cells from the periphery to the inflamed synovial tissue. The potential for CXCR3-mediated trafficking of transitional B cells to the site of inflammation after B cell depletion therapy and any possible contribution to disease amelioration warrant further examination.

### RA patient–derived B cells express high levels of proinflammatory cytokines under hypoxic conditions, mimicking the environment of the inflamed RA joint.

**Figure 4:**
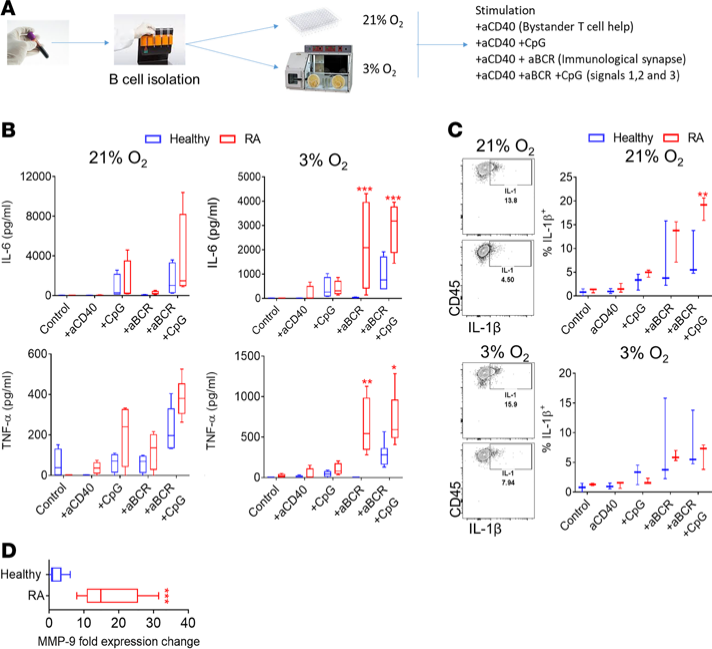
The effect of hypoxia on RA patient–derived B cells. (**A**) Schematic representation of peripheral blood B cell isolation and stimulation in vitro under normoxic or hypoxic conditions. aCD40, anti-CD40. (**B**) ELISA for the assessment of IL-6 and TNF-α concentration in HC- or RA patient–derived B cell cultures following stimulation as indicated under normoxic or hypoxic conditions (*n* = 4–6/group). Ordinary 2-way ANOVA with Holm-Šidák multiple-comparisons test was performed. *P < 0.05, **P < 0.01, ***P < 0.001. (**C**) Representative flow cytometric analysis plots and frequency of IL-1β–expressing HC- or RA patient–derived B cells following in vitro stimulation under the designated conditions (*n* = 3/group, 2 independent experiments). Ordinary 2-way ANOVA with Holm-Šidák multiple-comparisons test was performed. **P < 0.01. (**D**) Fold expression change of RA patient–derived B cell *MMP-9* expression compared with HC-derived B cells following stimulation under hypoxic conditions (*n* = 5/group). Statistical analysis was performed by using paired standard Student’s *t* test. *P < 0.05, ***P < 0.001. All data are represented as a box-and-whisker plot, with bounds from 25th to 75th percentile, median line, and whiskers ranging from 5th to 95th percentile.

Previous studies have shown a positive correlation between hypoxia and cellular infiltration of the RA synovial tissue (17, 18). We therefore examined the effect of hypoxic conditions that mimic the microenvironment of the inflamed joint on B cell activation and cytokine production. RA patient– or HC-derived B cells were isolated and cultured under atmospheric oxygen levels (normoxia) or 3% O_2_ (hypoxia), which is the previously estimated in vivo average oxygen concentration in the inflamed joints of RA patients (17, 18). The expression of TNF, IL-6, and IL-1β by RA- and HC-derived B cells stimulated under normoxic and hypoxic conditions was examined. Under normoxic conditions, there was increased (*P* = 0.0047) IL-1β production but not TNF or IL-6 by RA-derived, compared with HC-derived, B cells when stimulated via the B cell receptor (BCR) with additional TLR stimulation (Figure 4, A–C). Under hypoxic conditions that mimic the microenvironment of the inflamed joint, however, RA-derived B cells secreted significantly higher IL-6 and TNF when stimulated through the BCR (*P* = 0.0003, *P* = 0.007, respectively) with or without additional TLR stimulation (*P* = 0.0007, *P* = 0.03, respectively) (Figure 4, A–C). In addition to the increased proinflammatory cytokine production, under hypoxic conditions, RA patient–derived B cells showed significantly higher expression of MMP-9 (*P* = 0.005), which is indicative of increased invasive capacity compared with HC-derived B cells (Figure 4D). These data demonstrate the importance of oxygen availability for B cell stimulation and cytokine production and raise important considerations for the interpretation of in vitro data performed under atmospheric O_2_ conditions.

### Activated PD-1^+^ B cells accumulate at the site of inflammation in RA.

**Figure 5:**
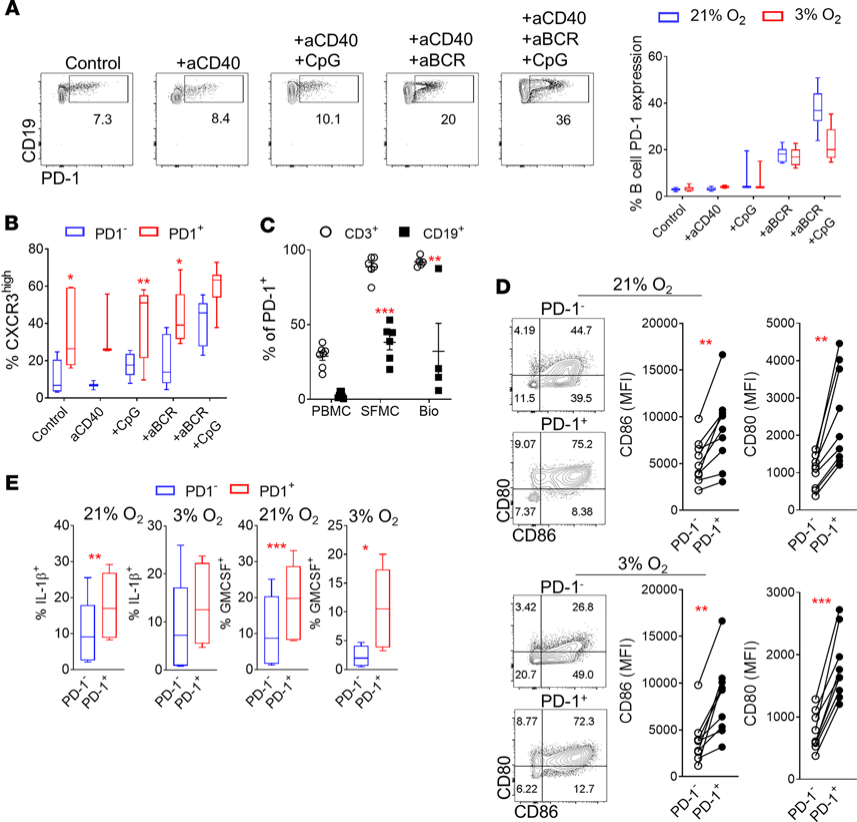
Identification of synovial PD-1^+^ B cells in RA. (**A**) Representative flow cytometric analysis and cumulative data for the identification of PD-1^+^ RA patient–derived B cells following in vitro stimulation under the indicated conditions (*n* = 3–6/group, 3 independent experiments). Data are represented as a box-and-whisker plot, with bounds from 25th to 75th percentile, median line, and whiskers ranging from 5th to 95th percentile. (**B**) Frequency of CXCR3 expression by PD-1^–^ and PD-1^+^ RA patient–derived B cells under the indicated conditions. *n* = 5–7/group and *n* = 3 for aCD40. Data are represented as a box-and-whisker plot, with bounds from 25th to 75th percentile, median line, and whiskers ranging from 5th to 95th percentile. Ordinary 2-way ANOVA with Holm-Šidák multiple-comparisons test was performed. *P < 0.05, **P < 0.01. (**C**) Frequency of RA patient peripheral blood (PBMC), SF (SFMC), and synovial tissue (Bio) PD-1–expressing CD3^+^ T cells and CD19^+^ B cells. *n* = 3–7/group. Each symbol represents an individual sample. Data are presented as mean ± SEM. Ordinary 2-way ANOVA with Holm-Šidák multiple-comparisons test was performed. **P < 0.01, ***P < 0.001. (**D**) Representative flow cytometric analysis plots and frequency of CD80 and CD86 expression by RA patient–derived B cells following in vitro stimulation (aCD40+aBCR+CpG) under normoxic or hypoxic conditions (*n* = 8, 3 independent experiments). Each symbol represents an individual sample. Paired Student’s *t* test was performed. **P < 0.01, ***P < 0.001. (**E**) Frequency of IL-1β– and GM-CSF–expressing PD-1^–^ and PD-1^+^ RA patient–derived B cells following stimulation under the indicated conditions (*n* = 6, 3 independent experiments). Data are represented as a box-and-whisker plot, with bounds from 25th to 75th percentile, median line, and whiskers ranging from 5th to 95th percentile. Statistical analysis was performed by using paired standard Student’s *t* test. *P < 0.05, **P < 0.01, ***P < 0.001.

Following in vitro stimulation by BCR-mediated signals, RA patient–derived B cells showed marked upregulation of PD-1 expression under normoxic or hypoxic conditions (Figure 5A). Earlier in this study, we demonstrated the importance of CXCR3 for the accumulation of memory B cells to the site of inflammation in RA. Importantly, the majority of CXCR3-expressing B cells following activation in vitro constitutively expressed PD-1 (Figure 5B). PD-1–expressing B cells constitute a rare (~2% of CD19^+^) population of cells in the periphery; however, there was significant accumulation of these cells in the SF (*P* = 0.0002) and synovial tissue (*P* = 0.005) in RA (Figure 5C). Immunofluorescence analysis of RA patient synovial tissue biopsies showed preferential accumulation of CD19^+^PD-1^+^ cells in tertiary lymphoid-like structures (Supplemental Figure 4). RA patient–derived PD-1–expressing B cells had higher expression of CD86 and CD80 compared with their PD-1^–^ counterparts under normoxic (*P* = 0.0026, *P* = 0.001, respectively) and hypoxic (*P* = 0.0015, *P* < 0.0001, respectively) conditions (Figure 5D). Importantly, PD-1^+^ RA patient–derived B cells maintained significantly higher expression of IL-1β under normoxic (*P* = 0.0031) conditions and GM-CSF under normoxic and hypoxic conditions (*P* = 0.0003, *P* = 0.013, respectively) compared with PD-1^–^ counterparts (Figure 5E).

Previous studies have shown a potential immunoregulatory effect of PD-1^+^ B cells in patients with thyroid cancer because of an increased expression of PD-L1 by these cells (19). PD-1^+^ RA patient–derived B cells stimulated in vitro showed similar PD-L1 expression levels compared with matched PD-1^–^ B cells (Supplemental Figure 5).

### PD-1^+^ RA patient B cells are dependent on glucose uptake and STAT3 activation.

**Figure 6:**
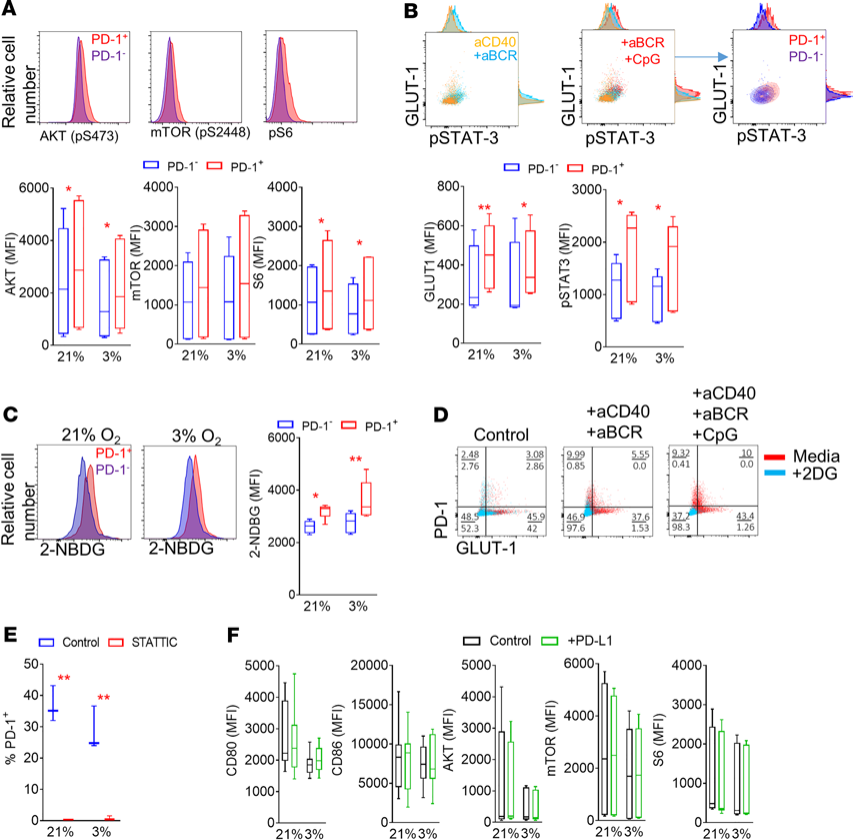
PD-1^+^ RA patient B cells are dependent on glycolysis. (**A**) Representative flow cytometric analysis histograms and cumulative MFI of RA patient peripheral blood–derived PD-1^–^ and PD-1^+^ B cell expression of phosphorylated AKT, mTOR, and S6 following stimulation (aCD40+aBCR+CpG) under normoxic (21% O_2_) and hypoxic (3% O_2_) conditions (*n* = 6/group, 3 independent experiments). Data are represented as a box-and-whisker plot, with bounds from 25th to 75th percentile, median line, and whiskers ranging from 5th to 95th percentile. (**B**) Representative flow cytometric analysis plots and MFI of RA patient–derived PD-1^–^ and PD-1^+^ B cell expression of GLUT1 and STAT3 phosphorylation (pSTAT3) following stimulation under the indicated conditions (*n* = 4/group, 2 independent experiments). Data are represented as a box-and-whisker plot, with bounds from 25th to 75th percentile, median line, and whiskers ranging from 5th to 95th percentile. (**C**) Representative flow cytometric analysis histograms of glucose analog 2-NBDG uptake by RA patient–derived PD-1^–^ and PD-1^+^ B cells under the indicated conditions, (*n* = 5/group, 2 independent experiments). Data are represented as a box-and-whisker plot, with bounds from 25th to 75th percentile, median line, and whiskers ranging from 5th to 95th percentile. Ordinary 2-way ANOVA with Holm-Šidák multiple-comparisons test was performed. *P < 0.05, **P < 0.01. (**D**) Representative flow cytometric analysis plots of PD-1 expression by RA patient–derived B cells following stimulation in the presence of glucose analog 2DG (*n* = 4, 2 independent experiments). (**E**) Frequency of PD-1 B cells following incubation with STAT3 small molecule inhibitor Stattic (*n* = 3). Data are presented as mean ± SEM. Ordinary 2-way ANOVA with Holm-Šidák multiple-comparisons test was performed. **P < 0.01. (**F**) Effect of PD-1/PD-L1 engagement on PD-1^+^ RA patient–derived B cell expression of CD80 and CD86 and phosphorylation of AKT, mTOR, and S6 under normoxic or hypoxic conditions (*n* = 9). Data are represented as a box-and-whisker plot, with bounds from 25th to 75th percentile, median line, and whiskers ranging from 5th to 95th percentile. Statistical analysis was performed by using paired standard Student’s *t* test.

PD-1 expression is dependent on BCR-mediated signals, with TLR9 activation enhancing that effect. Because of the dependency of PD-1 on BCR signaling, the activation of AKT, a downstream kinase of the BCR signaling cascade, was analyzed under normoxic and hypoxic conditions. RA patient–derived PD-1^+^ B cells expressed significantly (*P* = 0.011 normoxia, *P* = 0.022 hypoxia) higher levels of activated AKT compared with PD-1^–^ counterparts (Figure 6A) (20). AKT is a key regulator of mTOR. PD-1^+^ B cells showed higher activation of mTOR compared with PD-1^–^ B cells (Figure 6A) (21). To assess if the increased phosphorylation of mTOR translates to higher downstream activity, the phosphorylation of the ribosomal protein S6, a target of the mTOR pathway, was assessed. In agreement with the increased AKT/mTOR activity, PD-1^+^ B cells showed significantly (*P* = 0.023 normoxia, *P* = 0.031 hypoxia) higher S6 phosphorylation compared with PD-1^–^ B cells (Figure 6A).

The AKT/mTOR pathway has previously been shown to control glucose uptake and metabolism; therefore, we examined glucose transporter 1 (GLUT1) expression in RA patient PD-1^+^ and PD-1^–^ B cells (22, 23). BCR engagement led to increased B cell GLUT1 expression that correlates with STAT3 phosphorylation, indicating a potential association between BCR signaling strength and GLUT1 upregulation, while additional TLR9-dependent signals enhanced the BCR-mediated effect (Figure 6B). PD-1^+^ B cells had significantly (*P* = 0.002 and *P* = 0.015, respectively) higher expression of GLUT1 and phosphorylation of STAT3 (*P* = 0.014 and *P* = 0.021, respectively) than PD-1^–^ B cells under normoxic and hypoxic conditions (Figure 6B). The increased expression of GLUT1 led to significantly increased glucose uptake of PD-1^+^ compared with PD-1^–^ B cells, under normoxic (*P* = 0.002) and hypoxic (*P* = 0.015) conditions, as assessed by the incorporation of the fluorescent glucose analog 2-(*N*-(7-nitrobenz-2-oxa-1,3-diazol-4-yl)amino)-2-deoxyglucose (2-NBDG) (Figure 6C). Additional indication that PD-1–expressing RA patient B cells rely on glycolysis is the increased mitochondrial mass of these cells under normoxic and hypoxic conditions compared with their PD-1^–^ counterparts (Supplemental Figure 6). Deprivation of glucose by addition of the glucose analog 2-deoxyglucose (2DG) that fails to undergo glycolysis led to the elimination of PD-1–expressing B cells under in vitro stimulation conditions (Figure 6D). PD-1^+^ B cells had higher activation of STAT3 compared with PD-1^–^ B cells; therefore, we inhibited STAT3 activation using a small molecular weight inhibitor, Stattic (24). STAT3 inhibition led to complete loss of PD-1^+^ B cells under normoxic and hypoxic conditions (*P* = 0.0014, *P* = 0.004, respectively) (Figure 6E).

PD-1/PD-L1 interactions have been proposed to dampen down the BCR-mediated downstream signaling (25). Plate-bound recombinant PD-L1 was utilized to assess the effect of PD-1/PD-L1 engagement on the activation and metabolic status of PD-1^+^ B cells. PD-L1 did not result in decrease of CD80 and CD86 expression or AKT, mTOR, and S6 activation under normoxic or hypoxic conditions (Figure 6F).

### Altered cytokine, antigen presenting, and glycolysis gene signatures in ex vivo PD-1^+^ RA B cells.

**Figure 7:**
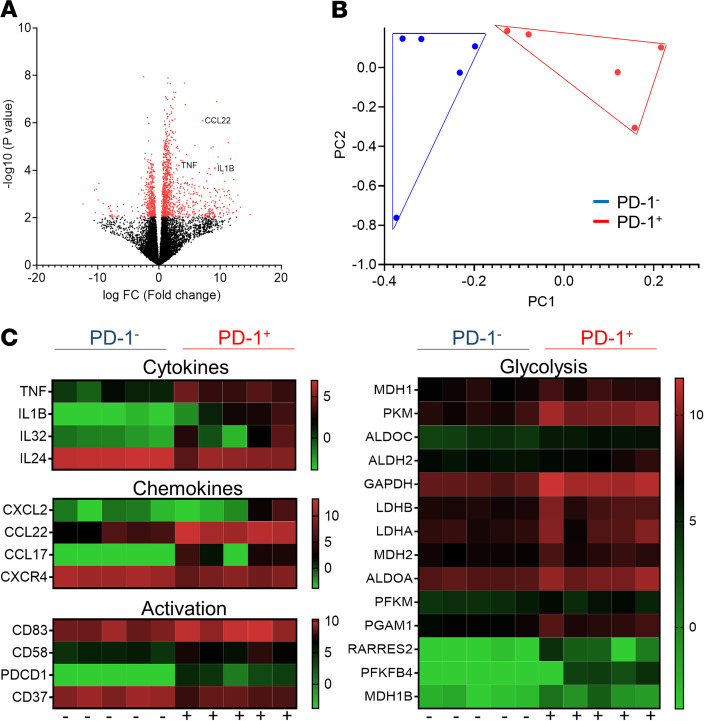
Differential gene expression of ex vivo RA patient PD-1^+^ and PD-1^–^ B cells. (**A**) Differential gene expression volcano plot of flow sorted, ex vivo RA patient–derived PD-1^+^ compared with PD-1^–^ B cells. Red-colored genes show significant differential expression between the 2 groups. (**B**) PCA analysis of flow sorted, ex vivo RA patient PD-1^+^ and matched PD-1^–^ B cells. Each point represents an independent sample. (**C**) Heatmap of RNA-Seq expression *Z*-scores for selected differentially regulated genes between PD-1^+^ and PD-1^–^ B cells. Each column represents an individual sample.

RNA-Seq analysis of ex vivo patient-derived B cells sorted on the basis of PD-1 revealed altered expression of approximately 900 genes between PD-1^+^ B cells and their PD-1^–^ counterparts (Figure 7A). Principal components analysis (PCA) of PD-1^+^ and PD-1^–^ B cells showed a separation between the 2 populations (Figure 7B). Proinflammatory cytokine gene expression for cytokines previously implicated in RA pathogenesis, namely *TNFA*, *IL6*, *IL1B*, and *IL32*, was significantly (*P* < 0.001) increased in PD-1^+^ B cells compared with PD-1^–^ B cells while the immunomodulatory *IL24* was significantly (*P* < 0.001) decreased (Figure 7C). Alterations were also observed in chemokine expression and genes involved in B cell maturation and activation, with PD-1^+^ B cells adopting an overall more activated profile than PD-1^–^ B cells (Figure 7C). Several genes involved in glycolysis were significantly upregulated in PD-1^+^ B cells in contrast to PD-1^–^ B cells, with pathway analysis showing enrichment in the glycolysis (*P* = 4.3 × 10^–9^), gluconeogenesis (*P* = 8.7 × 10^–7^), and oxidative phosphorylation (*P* = 4 × 10^–40^) pathways (Figure 7C, Supplemental Figure 7). To complement this analysis, a gene set enrichment analysis (GSEA) was performed on the differentially expressed genes ranked by their fold change values, using the Hallmark gene signature from the Broad Institute’s Molecular Signatures Database. This analysis showed an enrichment in the glycolysis gene signature (normalized enrichment score [NES] = 1.37, FDR = 0.222) and a stronger enrichment in genes corresponding to inflammatory response (NES = 1.77, FDR = 0.008) and TNF-α signaling through NF-κB (NES = 1.60, FDR = 0.018).

### RA patient PD-1^+^ B cells are more glycolytic than their PD-1^–^ counterparts.

**Figure 8:**
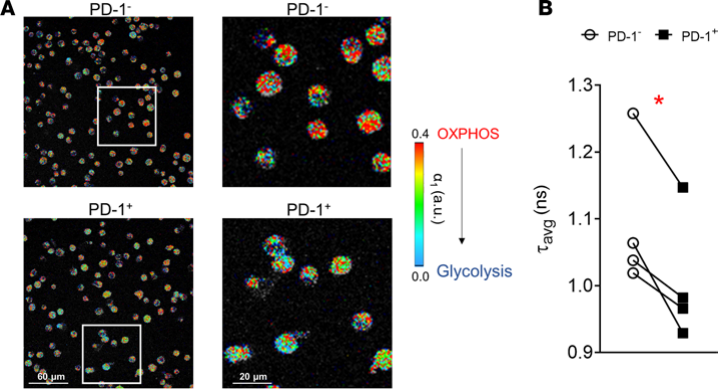
FLIM analysis of PD-1 B cell metabolic profile. (**A**) Representative multiphoton microscopy FLIM analysis of flow sorted RA patient–derived PD-1^–^ and PD-1^+^ B cells (4 independent experiments were performed). (**B**) Average PD-1^–^ and PD-1^+^ B cell emission lifetime (τ_avg_) of NAD following excitation. *n* = 4/group. Data are presented as mean ± SEM. A reduction in τ_avg_ is reflected in an increase in free NAD and therefore increased glycolysis. Statistical analysis was performed by using paired standard Student’s *t* test. *P < 0.05.

The data presented herein suggest an increased glycolytic capacity and glucose dependency of PD-1^+^ compared with PD-1^–^ RA patient–derived B cells. In order to directly assess whether PD-1^+^ B cells are more reliant on either glycolysis or oxidative phosphorylation (OXPHOS), FLIM was utilized. FLIM is based on the principle of endogenous fluorescence molecules such as NAD and as a result requires no staining, fixation, or other type of processing of the target cells (26). FLIM’s capacity to distinguish between bound and unbound NAD is based on the self-quenching ability of NAD. In unbound NAD the nicotinamide and adenine rings are in close proximity, and the fluorescent signal decay following excitation is approximately 0.4 ns; however, NAD in its bound form has a significantly lower signal decay in the range of 2 ns because of stretching of the molecule, leading to longer distance between the nicotinamide and adenine rings and reduced self-quenching (26, 27). Importantly, higher unbound to bound NAD ratio is directly proportionate to the cell’s glycolytic versus oxidative metabolic capacity. Visualization of NAD by FLIM offers direct evidence of the cell’s metabolic state. FLIM analysis of RA patient–derived PD-1^+^ sorted B cells revealed a significant (*P* = 0.022) preference for glycolysis compared with matched PD-1^–^ B cells (Figure 8, A and B).

## Discussion

B cell depletion therapy has been efficacious for the treatment of patients with RA. However, opportunities for more targeted therapeutic intervention that can minimize potential side effects should be explored. Herein, we demonstrate a role for CXCR3 in the accumulation of switched and DN memory B cells at the site of inflammation in RA. Peripheral blood CXCR3-expressing B cells closely mirrored the B cell subpopulation distribution in the inflamed RA joint with overrepresentation of switched memory and DN memory B cells. A negative correlation between the frequency of CXCR3-expressing B cells and disease activity in RA patients, potentially because of increased migration of peripheral blood CXCR3-expressing B cells to the site of inflammation in RA patients with increased disease severity, further reinforces the contribution of CXCR3 in the migration of activated memory B cells and raises the possibility for early therapeutic intervention by inhibiting the CXCR3-mediated synovial migration of memory B cells.

We have previously demonstrated that the RA joint is hypoxic with an average oxygen level of 3% and a positive correlation between hypoxia and synovial tissue cellular infiltration (17, 18). The effect of hypoxia on B cell function has not been fully elucidated; therefore, we examined the activation and costimulatory potential of RA patient–derived B cells, stimulated with physiologically relevant conditions under hypoxia, and identified a superior capacity of these cells to maintain proinflammatory cytokine production in a hypoxic environment recapitulating the inflamed joint. While the effect of hypoxia on RA B cell proinflammatory cytokine production was limited, with RA patient–derived B cells maintaining their capacity to produce proinflammatory cytokines, hypoxic conditions led to a reduction of proinflammatory cytokine production by HC-derived B cells. Therefore, hypoxia exacerbated the differences between HC- and RA patient–derived B cells. Additionally, RA patient–derived B cells cultured under hypoxic conditions expressed high levels of MMP-9 compared with HC-derived B cells; B cell MMP-9 expression has previously been correlated with clinical relapse in patients with multiple sclerosis and the capacity of B cells to invade the blood-brain barrier (28). In this study we identified a population of CXCR3^hi^PD-1^+^ B cells that preferentially accumulated in the synovial tissue in RA, as opposed to the peripheral blood, and were characterized by a markedly increased capacity for costimulation and proinflammatory cytokine production. PD-1^+^ B cells showed a strong dependency on glucose uptake and STAT3 activation and, based on direct visualization of bound and unbound forms of NAD by the potentially novel FLIM, were more glycolytic than their PD-1^–^ counterparts. Altered gene expression in over 900 genes was observed between PD-1^+^ and PD-1^–^ ex vivo patient B cells, with genes involved in B cell activation and proinflammatory cytokine production being upregulated in PD-1^+^ B cells compared with PD-1^–^ B cells. Additionally the glycolysis, gluconeogenesis, and oxidative phosphorylation pathways were enriched in PD-1–expressing B cells.

Several studies have previously demonstrated the capacity of B cells to express the T cell–associated coinhibitory factor PD-1. There is, however, a paucity of information on the functional effects of B cell PD-1 expression. A proposed mechanism of action for B cell PD-1 expression is the dampening of BCR-mediated downstream signaling, leading to decreased B cell activation and cytokine production (25, 29). Recent studies identify potentially tumorigenic B cells that express PD-1 and promote immune system regulation and tumor survival; however, there are discrepancies regarding the suggested mechanisms that these cells employ to exert their immunosuppressive effects. A study in patients with hepatoma shows PD-1^+^ B cell IL-10–dependent immune suppression of antitumor T cell responses, while a recent study in thyroid cancer patients highlights high PD-L1 expression and not IL-10 production by tumor PD-1 B cells as being responsible for T cell suppression and cancer survival (19, 30). An alternative suggested mechanism of action of B cell PD-1 that warrants further investigation is the possibility that PD-1 engages PD-L1 on the same cell in *cis* formation, resulting in reduced availability of PD-L1 for suppression of T cell activation during immunological synapse formation (31). We examined PD-L1 expression by RA patient–derived PD-1^+^ and PD-1^–^ B cells and observed no differences in their capacity to express PD-L1. Although PD-1^+^ B cell IL-10 secretion was not assessed, these cells were more activated, evidenced by increased expression of CD86 and CD80, and produced high levels of several proinflammatory cytokines, including GM-CSF. Pathogenic GM-CSF–expressing B cells have recently been described in humans. GM-CSF expression is increased following in vitro BCR-mediated stimulation in the presence of surrogate T cell help, with multiple sclerosis patient–derived B cells showing significantly higher GM-CSF–secreting capacity than HC-derived B cells (32). The proinflammatory characteristics of RA patient–derived PD-1^+^ B cells were evident under normoxic, and more notably, under hypoxic conditions that more closely resembled the unique environment of the inflamed RA joint.

RA patient PD-1^+^ B cells had higher activation of the AKT/mTOR/S6 pathway, leading to increased GLUT1 expression and glucose uptake under normoxic and hypoxic conditions compared with PD-1^–^ counterparts. Deprivation of glucose led to loss of PD-1 expression, and STAT3 activation coupled with direct visualization of NAD in RA B cells revealed a potent glycolytic profile, delineating an important role of glycolysis for the maintenance of PD-1^+^ B cells. There is a paucity of data on the metabolic requirement of B cell activation and function, and the effect of glycolysis on B cell biology has only recently been explored. B cells, following stimulation, rapidly increased glycolysis in a GLUT1-dependent manner, paralleled with increased antibody-producing capacity (33). Humoral responses can be greatly influenced by changes in oxygen availability, with activated B cells becoming more glycolytic under hypoxic conditions (34). Importantly, B cells exposed to chronic B cell–activating factor and autoimmune-prone B cells maintain high glycolytic capacity, with deletion of Glut-1 leading to reduced B cell proliferation and impaired antibody production (33). The increased glycolytic capacity of PD-1 B cells could in addition to their activation/proliferation/cytokine secretion also affect the metabolites they are contributing to their microenvironment. Further studies analyzing the metabolite contribution and cytokine production of PD-1 B cells are required.

While B cell infiltration of the joint correlates with disease activity and B cell depletion therapy leads to disease amelioration in autoantibody-positive and autoantibody-negative RA patients, B cells are a relatively small population of the immune infiltrate of the joint (5, 6). Further investigation of the role of B cells in synovitis and the particular role of RA joint PD-1 B cells is required. Additionally, a more expansive RA patient synovial biopsy sample size, inclusive of RA patients with high disease activity and paralleled with classification based on type of synovial infiltrate, would allow for the identification of relations between degree of PD-1 B cell infiltration, type of infiltrate, and synovitis.

In conclusion, we provide evidence in support of early therapeutic intervention by inhibiting the role of CXCR3 in B cell migration to the synovial tissue and the potential for more specific B cell therapeutic targeting of pathogenic, glycolytic PD-1–expressing synovial B cells. We also highlight the importance of careful data extrapolation from normoxic to more physiologically relevant hypoxic conditions that closely resemble the unique environment of the inflamed joint.

## Methods

### Synovial tissue single-cell suspensions.

Synovial biopsies (~15) were enzymatically and mechanically digested using the gentleMACS Tumor Dissociation Kit, human (Miltenyi Biotec), as per manufacturer’s instructions. Briefly, 15 synovial biopsies were placed in 4.7 mL of RPMI supplemented with 200 μL of enzyme H, 100 μL enzyme R, and 25 μL enzyme A in a gentleMACS C Tube followed by initial mechanical disruption of the tissue using program h_tumor_01 on a gentleMACS Dissociator. Samples were then incubated for a total of 1 hour at 37°C under continuous rotation using the MACSmix Tube Rotator with further applications of the gentleMACS Dissociator at the halfway point and at the end of the 1 hour incubation according to the manufacturer’s instructions. A single synovial cell suspension was generated by filtration through a 70 μm cell strainer. Matched PBMCs and SFMCs were also isolated using a density gradient preparation for direct comparison of B cell frequency in the circulation versus the inflamed synovium.

### Cell isolation and culture.

B cells were isolated by magnetic bead cell sorting using negative (human B Cell Isolation Kit II, Miltenyi Biotec) or positive selection on the basis of CD19 expression (CD19 MicroBeads, human, Miltenyi Biotec) according to the manufacturer’s instructions. Purity of the isolated B cells was routinely checked by flow cytometric analysis for the detection of the B cell marker CD20 and was over 90%. Isolated B cells were then cultured in cRPMI (RPMI from Glutamax, Thermo Fisher Scientific), supplemented with 10% FBS (MilliporeSigma) and 1000 U/mL pen/strep (MilliporeSigma) in a 5% CO_2_ humidified incubator at 37°C under atmospheric O_2_ conditions or a humidified hypoxia chamber (5% CO_2_, 37°C) at 3% O_2_ as indicated. Cells were left unstimulated or were stimulated in vitro for 72 hours (at 1 × 10^6^ cells/mL) with combinations of aCD40 (5 μg/mL, G28.5, *InVivo*MAb, Bio X Cell), F(ab′)_2_ aIgG + aIgM H and L chain cross-linking antibody (1 μg/mL, catalog 16-5099-85, Thermo Fisher Scientific), and CpG ODN2006 (0.2 μM, InvivoGen). Following incubation, supernatants were collected for cytokine analysis by ELISA, and cells were either analyzed by flow cytometric analysis or flow sorted (4-laser BD Aria flow sorter) on the basis of PD-1 expression for subsequent FLIM analysis.

### Flow cytometric analysis.

Isolated and in vitro–cultured HC and RA patient–derived B cells, PBMCs, SFMCs, and synovial tissue single-cell suspensions were subjected to flow cytometric analysis. The generation of synovial tissue single-cell suspension was performed as previously described (18). Briefly, approximately 15 synovial biopsies per patient were enzymatically and mechanically digested using the gentleMACS dissociation kit (Miltenyi Biotec) as per manufacturer’s instructions. Cells were then passed through a 70 μm cell strainer before analysis. All extracellular targets were evaluated for loss of expression due to enzymatic digestion. Of all the targets tested, only CD27 was cleaved and expression was artificially lost. However, incubation of the synovial tissue single-cell suspensions for 6 hours postdigestion restored CD27 B cell expression (Supplemental Figure 1). Following the generation of single-cell suspensions, cells were washed in PBS and incubated with LIVE/DEAD fixable NIR (Thermo Fisher Scientific) viability reagent as per the manufacturer’s instructions. Cells were then incubated with TruStain FcX receptor blocking solution (BioLegend) to minimize nonspecific antibody binding. Next, cells were stained with antibody combinations targeting surface markers for 30 minutes at 4°C (Supplemental Table 1). Following incubation, cells were washed twice in FACS buffer (PBS with 2% FBS and 0.002% *w/v* sodium azide). If intracellular staining was required, cells were subsequently fixed and permeabilized using the intracellular Foxp3 staining kit (eBioscience, Thermo Fisher Scientific) as per the manufacturer’s instructions. Briefly, following incubation with the kit’s fix/perm buffer at 4°C for 30 minutes, cells were washed in perm buffer and incubated with antibody combinations for intracellular staining for 30 minutes at 4°C (Supplemental Table 1). Cells were then washed once with perm buffer and once with the FACS buffer before acquisition on a 4-laser LSRFortessa cytometer (BD).

### Glucose uptake and mitochondrial mass analysis.

Sorted B cells were cultured in vitro under normoxic or hypoxic conditions and stimulated as described herein. For the last 30 minutes of culture, the cell culture medium was replaced with glucose-free RPMI (Thermo Fisher Scientific) supplemented with 1000 U/mL pen/strep (MilliporeSigma) and 50 μM of 2-NBDG (Invitrogen, Thermo Fisher Scientific). Prior to addition of the glucose-free cell culture medium, the media were left to equilibrate under the respective normoxic or hypoxic conditions of the cells. Following incubation, cells were washed in PBS, incubated with viability dye followed by Fc blocking step and extracellular staining as described herein, and then immediately acquired on a 4-laser Fortessa analyzer (BD). Relative mitochondrial mass was estimated based on incorporation of MitoTracker Green (Thermo Fisher Scientific). RA patient–derived B cells were isolated and stimulated under normoxic and hypoxic conditions as described herein. During the last 30 minutes of culture, cells were washed and resuspended in RPMI without FBS supplemented with 20 nM of MitoTracker Green. Cells were washed and stained for viability and expression of PD-1 before acquisition on a 4-laser Fortessa analyzer.

### Immunofluorescence.

Synovial biopsies were fixed in 10% neutral-buffered formalin solution followed by paraffin embedding. Synovial tissue sections, 3 μm thick, were heated for 30 minutes at 60°C, deparaffinized in xylene, and rehydrated in alcohol and deionized water. Antigen retrieval was performed by heating sections in antigen retrieval solution (15 mL of 1 M sodium citrate and 15 mL of 1 M citric acid in deionized water, pH 6.0) in a pressure cooker. Slides were washed in PBS for 5 minutes. Nonspecific binding was blocked using 10% casein in PBS for 30 minutes. Primary antibodies aPD-1 (Abcam, clone: NAT105) and aCD19 (Thermo Fisher Scientific, clone: JF100-06) were incubated on sections for 2 hours at room temperature. An IgG1 control antibody (Dako) was used as a negative control. Slides were washed in PBS/Tween followed by 1-hour incubation at room temperature with secondary Cy2 (catalog 115-225-146) and Cy3 (catalog 111-165-144) AffiniPure secondary antibodies (Jackson ImmunoResearch). Slides were washed with PBS/Tween and PBS, before counterstaining of nuclei with DAPI (MilliporeSigma) and cover slide mounting with ProLong Gold Antifade (Thermo Fisher Scientific). Stained cells were visualized with a Leitz DM40 microscope (Leica Microsystems), and images were captured using the AxioCam system and AxioVision 3.0.6 software (Carl Zeiss Inc).

### ELISA.

Tissue culture supernatants of sorted and in vitro–cultured B cells were analyzed by ELISA for the presence of IL-6 (DuoSet, R&D, Bio-Techne) or TNF-α (DuoSet, R&D Systems, Bio-Techne) according to the manufacturer’s instructions.

### PCR.

RA patient and HC peripheral blood B cells were isolated and cultured under normoxic or hypoxic conditions as described herein. Total RNA was isolated using the RNeasy Mini Kit (QIAGEN) according to the manufacturer’s instructions. RNA quantification was performed on a NanoDrop spectrophotometer (Thermo Fisher Scientific). Samples with a 260/280 nm and 260/230 nm ratio of 1.8 or above were used for subsequent cDNA synthesis. Total RNA was reverse-transcribed to cDNA using the RT2 First Strand Kit (QIAGEN) as per manufacturer’s instructions. Genomic DNA elimination was performed as described previously (19). PCR was performed using the RT2 SYBR Green Mastermix (QIAGEN) as per the manufacturer’s instructions. PCR was performed on a LightCycler 480 System (Roche Diagnostics) with the following primers: MMP9 forward: ATTGGATCCAAAACTACTCGGAAGA, MMP9 reverse: GGGCAAAGGCGTCGTCAATC. Relative gene expression changes were determined by the 2−ΔΔCt method and normalized to the housekeeping gene RPLPO (primers: forward: GCGTCCTCGTGGAAGTGACATCG and reverse: TCAGGGATTGCCACGCAGGG) (all from Microsynth).

### Cell invasion assays.

RA patient–derived B cells, isolated as described herein, were seeded at a density of 1.5 × 10^4^ cells per well with aCD40 (5 μg/mL) in the migration chamber of a 24-well Corning Matrigel (8 μm membrane precoated with Matrigel, Thermo Fisher Scientific) with or without addition of the small molecular weight antagonist of CXCR3, AMG487 (200 μM, Tocris, Bio-Techne). Medium supplemented with or without 10% RA patient ex vivo synovial biopsy-conditioned media was added to the well. The cells were incubated for 24 hours. Noninvading cells remaining on the migration chamber were removed and counted by flow cytometric analysis using cell counting beads (CountBright absolute counting beads, Thermo Fisher Scientific) following LIVE/DEAD and CD20 staining. Cells that migrated from the insert to the corresponding well were similarly counted by flow cytometric analysis.

### Fluorescence lifetime imaging microscopy.

Peripheral blood RA patient–derived B cells, isolated as described above, were stimulated in vitro for 72 hours with aCD40 (5 μg/mL), F(ab′)_2_ aIgG + aIgM H and L chain cross-linking antibody (1 μg/mL), and CpG ODN2006 (0.2 μM). Cells were cultured at 1 × 10^6^ cells/mL. B cells were then flow sorted with a purity greater than 98% on the basis of PD-1 expression using a 4-laser Aria sorter (BD) and were immediately transferred to an 18-well 15-μ-Slide (Ibidi). FLIM utilizes endogenous fluorophores such as NAD in order to obtain an image. FLIM’s capacity to distinguish between bound and unbound NAD is based on the self-quenching process of NAD. In unbound NAD, the nicotinamide and adenine rings are in close proximity, and the fluorescence decay signal following excitation is approximately 0.4 ns. When in protein-bound form, NAD has a significantly longer fluorescence lifetime in the range of 2–4 ns due to stretching of the molecule, leading to longer distance between the nicotinamide and adenine rings and reduced self-quenching (20–22). FLIM was performed using an upright Olympus BX61W1 multiphoton microscopy system equipped with a Ti:Sapphire Laser (Chameleon Ultra, Coherent), a water-immersion objective (×25 Olympus 1.05 NA), and a temperature-controlled stage (37°C). NAD excitation was performed at a wavelength of 760 nm, and fluorescence emission was selected with a 455/90 nm bandpass filter. Fluorescence decay measurements were obtained using a PicoHarp 300 TCSPC system operating in the time-tagged mode coupled with a photomultiplier detector assembly hybrid detector (PicoQuanT GmbH) at 256 time bins per pixel.

A 2-component fitting was used to differentiate between the free (τ_1_) and protein-bound (τ_2_) NAD(P)H: the average lifetime (τ_avg_) of NAD(P)H for each pixel was calculated by a weighted average of both free and bound lifetime contributions: τ_avg_ = ([α_1_ × τ_1_] + [α_2_ × τ_2_])/(α_1_ + α_2_).

### RNA-Seq.

Single-end 75-bp RNA-Seq at a read depth of 50,000,000 reads per sample was performed. Raw reads from FASTQ files were assessed for quality using FastQC. The reads were then pseudoaligned and transcript expression quantified (35) with *kallisto*. Transcripts were annotated with the *Homo sapiens* Ensembl version 86 build. The resulting transcript counts were used for differential analysis with edgeR after removal of transcripts with low counts. Testing for differential expression was performed using the generalized linear model options in edgeR (glmQFTest function in the Bioconductor *edgeR* package). Our RNA-Seq data have been deposited in the NCBI’s Gene Expression Omnibus database (accession number GSE154988). PCA plots of the samples based on their transcript profiles were plotted using the R *stats* and *ggplot2* packages. Downstream pathway enrichment studies were performed using a variety of methods, including GSEA and gene set variation analysis as implemented in the corresponding R/Bioconductor package.

Further information can be found in Supplemental Methods.

### Statistics.

Statistical analysis was performed using Prism 7 (GraphPad) software. One-way or 2-way ANOVA with Tukey’s multiple-comparisons test and unpaired 2-tailed standard Student’s *t* test was used as indicated. Statistical significance was considered with *P* values of less than 0.05.

### Study approval.

Peripheral blood, SF, and synovial tissue samples were collected from patients who were recruited from the Rheumatology Department, St. Vincent’s University Hospital; University College Dublin; and Tallaght University Hospital, Trinity College Dublin (Supplemental Table 2 and Supplemental Table 3). HC peripheral blood samples were obtained from buffy coats from St. James’s Hospital blood transfusion department and healthy volunteers recruited at Trinity Biomedical Sciences Institute and St. Vincent’s University Hospital. All subjects gave fully informed written consent approved by the institutional Ethics Committee, and research was performed in accordance with the Declaration of Helsinki. RA patient arthroscopies were performed under local anesthetic using Wolf 2.7 mm needle arthroscopy or ultrasound-guided biopsy as previously described (17).

## Author contributions

AF designed and performed experiments, analyzed data, and wrote the manuscript. NN performed FLIM assay and analyzed data. VM performed experiments. KM recruited patients and obtained patient samples. BM performed flow cytometric cell sorting. MGM analyzed data and wrote the manuscript. CL recruited patients and obtained patient samples. RHM recruited patients and obtained patient samples. NR analyzed data. VK performed RNA-Seq data analysis and wrote the manuscript. SN analyzed data and wrote the manuscript. DJV recruited patients and obtained patient samples, analyzed data, and wrote the manuscript. UF designed experiments, analyzed data, and wrote the manuscript.

## Supplementary Material

supplemental data

## Version 1 11/05/2020

Electronic publication

## Version 2 11/10/2020

Updated Acknowledgments
