# Supplementary material for: Pathogenic, glycolytic PD-1+ B cells accumulate in the hypoxic RA joint
Source: JCI Insight. 2020 Nov 5;5(21):e139032. doi: 10.1172/jci.insight.139032 (PMC7710281; doi:10.1172/jci.insight.139032)
Supplement: supplemental data [file jciinsight-5-139032-s240.pdf]

Sup. Figure 1

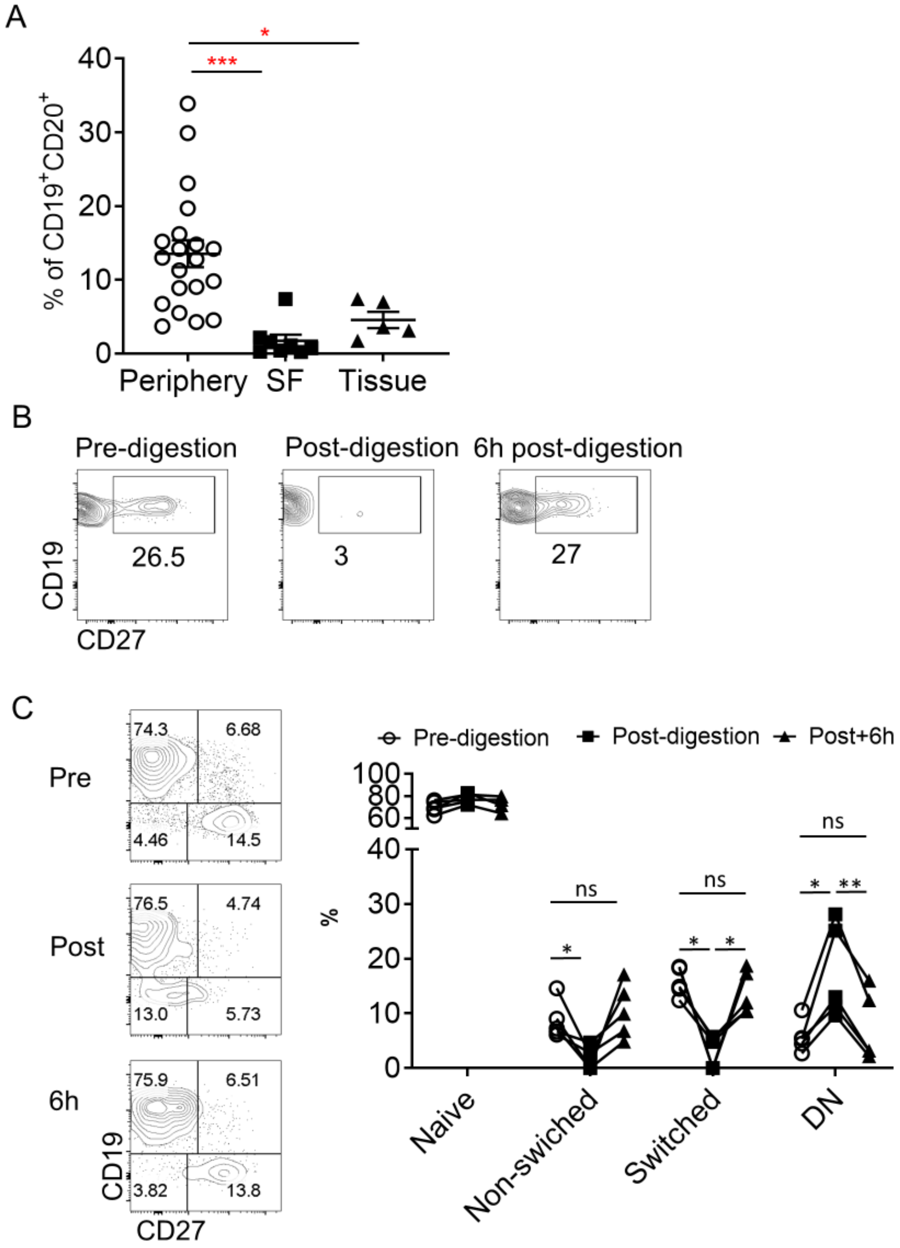

Supplementary Figure 1. RA patient synovial tissue and peripheral blood B cell frequency analysis.

(A). Frequency of $CD19^{+}CD20^{+}$ B cells in the periphery (n=20), synovial fluid (n=8) and synovial tissue (n=5) of RA patients. (B). Representative analysis of CD27 expression by B cells pre-, post- and 6 hours post-enzymatic digestion.

Sup. Figure 2

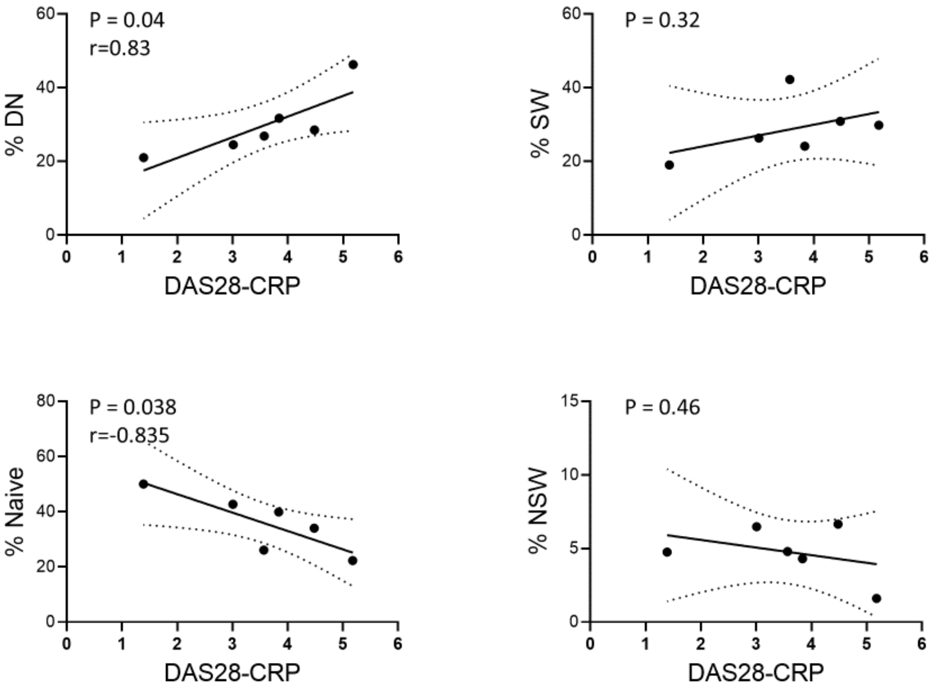

Supplementary Figure 2. RA synovial tissue accumulation of memory B cells.

Correlations of RA patient synovial tissue B cells and corresponding patient's DAS18-CRP. Each symbol represents an individual sample, 95% confidence bars and best fit line are shown, Pearson correlation coefficients were calculated and p<0.05* were considered significant.

Sup. Figure 3

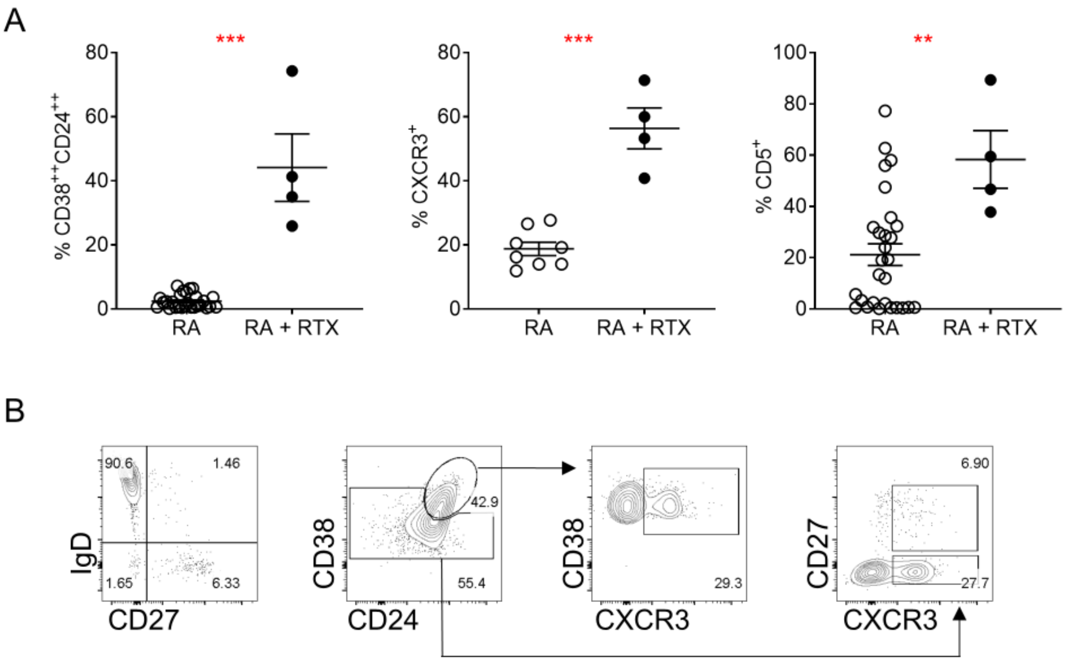

Supplementary Figure 3. Expression of CXCR3 by returning RA patient B cells following depletion with rituximab.

Frequency of transitional ($CD38^{++}CD24^{++}$), $CXCR3^{+}$ and $CD5^{+}$ B cells returning 6 to 12 months following Rituximab infusion (n=4) and non-Rituximab treated RA patient controls (n=8-28). All data are presented as mean ± SEM, each symbol represents an individual sample. Statistical analysis was performed by using two tailed standard Student's t test, $p<0.05$* were considered significant.

Sup. Figure 4

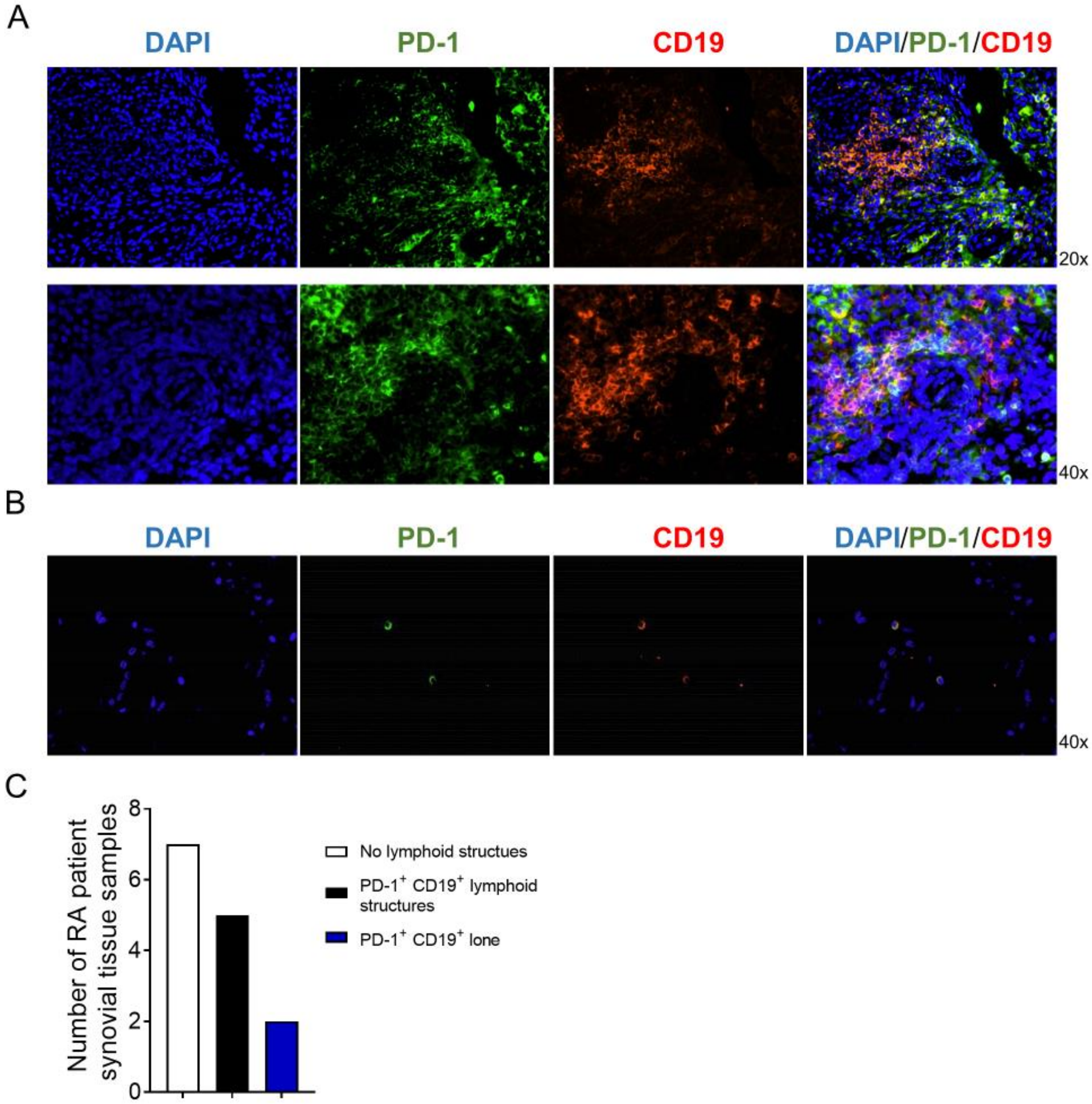

Supplementary Figure 4. RA patient synovial tissue localisation of PD-1^{+} B cells.

Immunofluorescence of RA patient synovial tissue samples for the identification of CD19^{+} and PD-1^{+} expressing cells. (A). Representative examples of CD19^{+} and PD-1^{+} expressing cells located in tertiary lymphoid organ-like formations. (B). Representative example of lone CD19^{+} and PD-1^{+} expressing cells in RA patient synovial tissue. (C). Summary of tissue samples analysed and distribution of CD19^{+}PD-1^{+} cells. A total of 13 RA patient synovial tissue samples were analysed, 5 showed accumulation of CD19^{+}PD-1^{+} in lymphoid like structures, 2 showed lone CD19^{+}PD-1^{+} cells and 7 showed no lymphoid like structure formation or CD19^{+} cell synovial infiltration.

Sup. Figure 5

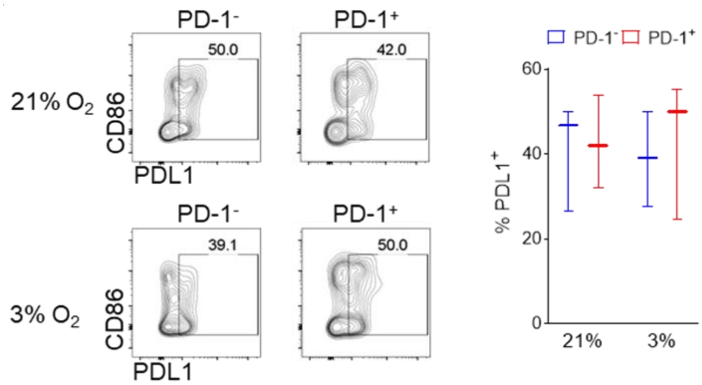

Supplementary Figure 5. PDL1 expression by *in vitro* stimulated PD-1^{+} and PD-1^{-} RA patient derived B cells.

Representative flow cytometric analysis plots and frequency for the expression of PDL1 by PD-1^{+} and PD-1^{-} RA patient derived B cells following stimulation under normoxic or hypoxic conditions (n=3/group, two independent experiments) data are represented as a box-and-whisker plot, with bounds from 25th to 75th percentile, median line, and whiskers ranging from 5th to 95th percentile.. Statistical analysis was performed by using paired standard Student's t test, p<0.05* were considered significant.

Sup. Figure 6

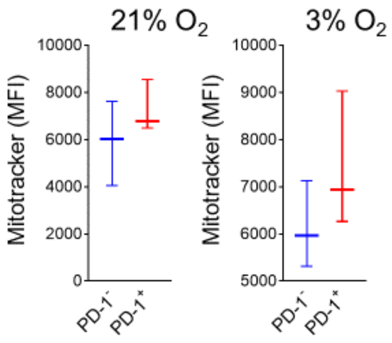

Supplementary Figure 6. RA patient PD-1$^-$ and PD-1$^+$ B cell mitochondrial mass comparison.

Mittotracker MFI following flow cytometric analysis of RA patient derived PD-1$^-$ and PD-1$^+$ B cells following stimulation under normoxic or hypoxic conditions (n=3/group, two independent experiments) data are represented as a box-and-whisker plot, with bounds from 25th to 75th percentile, median line, and whiskers ranging from 5th to 95th percentile.. Statistical analysis was performed by using paired standard Student's t test, p<0.05* were considered significant.

Sup. Figure 7

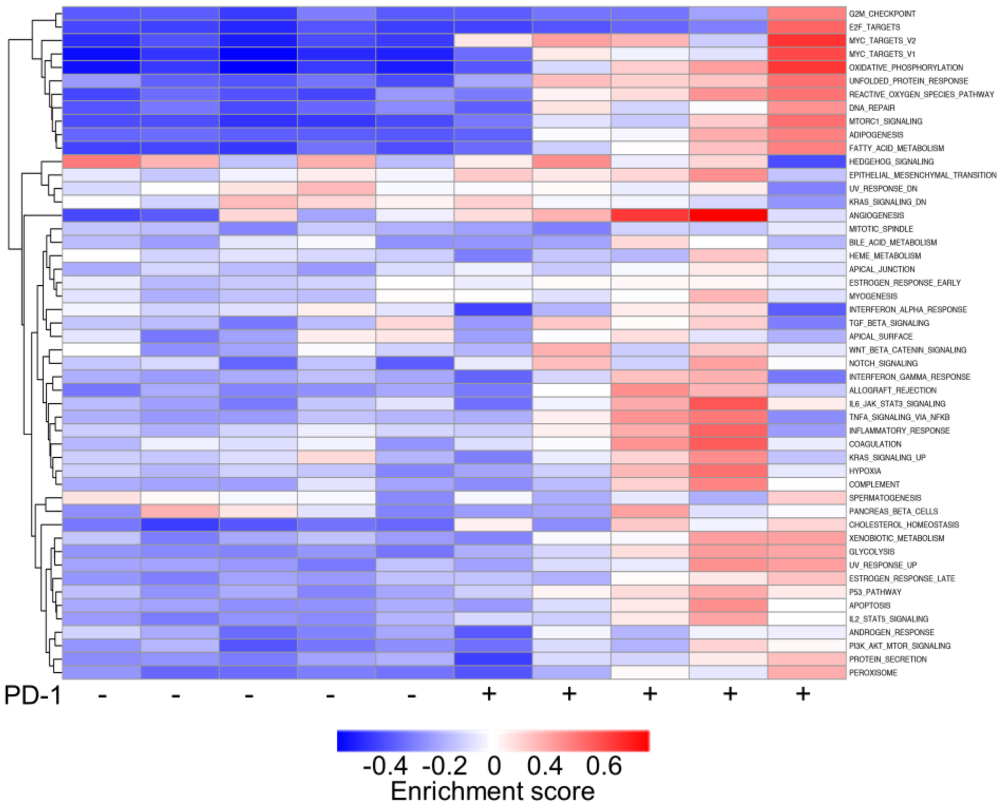

Supplementary Figure 7. RNAseq pathway enrichment analysis of RA patient paired PD-1^{+} and PD-1^{-} B cells.

Flow sorted, *ex vivo* RA patient PD-1^{+} and matched PD-1^{-} B cells were subjected to RNAseq analysis followed by pathway analysis. Pathway analysis was performed by processing the list of differentially expressed genes using the Ingenuity Pathway Analysis (IPA) software (Qiagen). Only genes that differentially expressed with an FDR value of < 0.05 were used.

Target	Conjugate	Clone	Supplier
CD38	Alexa Fluor 488	HIT2	BioLegend
CD24	PerCP-Cy5.5	ML5	BD Biosciences
CD20	APC	2H7	BD Biosciences
CD80	APC-R700	307.5	BD Biosciences
CD27	Brilliant Violet 421	O323	BioLegend
IgM	Brilliant Violet 510	G20-127	BD Biosciences
CD138	Brilliant Violet 605	MI15	BioLegend
CD45	Brilliant Violet 650	H130	BioLegend
CD19	Brilliant Violet 711	HIB19	BioLegend
MHC class II	Brilliant Violet 786	G46-6	BD Biosciences
CD269 (BCMA)	PE	19F2	BioLegend
CD40	PE-CF594	5C3	BioLegend
CD86	PE-Cy5	IT2.2	BD Biosciences
IgD	PE-Cy7	1A6-2	BioLegend
PD-1	BV421	NAT105	BioLegend
PD-1	PE	NAT105	BioLegend
CD45	PE-Cy5	H130	BioLegend
CD45	BV510	H130	BioLegend
GLUT1	PE	202915	Bio-Techne
CXCR3	PE	G025H7	BioLegend
CXCR3	BV650	G025H7	BioLegend
CD3	BV786	HIT3a	BioLegend
CCR7	PE-CF594	G043H7	BioLegend
CD8a	PE-Cy5	HIT8a	BioLegend
CD4	PE-Cy7	RPA-T4	BioLegend
CCR6	BV711	G034E3	BioLegend
CXCR5	BV785	J252D4	BioLegend
pSTAT3	PE-CF594	4/P-STAT3	BD
PDL1	BV650	MIH1	BD

IL-1β	PE	CRM56	Thermofisher
AKT	PD-CF594	M89-61	BD
mTOR	AF647	O21-404	BD
S6	AF488	N7-548	BD

Supplementary table 1: Characteristics of antibodies used for flow cytometric analysis. Sample acquisition was performed on a 4-laser BD LSR Fortessa II cell analyser.

Figure and sample	CRP	ESR	SJC28	TJC28	VAS	DAS28
**Figure 1F (1)**	19.7	30	2	0	40	3.01
**Figure 1F (2)**	<1	-	2	2	100	3.57
**Figure 1F (3)**	53.6	98	4	6	60	5.18
**Figure 1F (4)**	8	43	0	3	80	3.84
**Figure 1F (5)**	<1	2	1	0	10	1.39
**Figure 1F (6)**	10.4	3	0	12	50	4.48
**Figure 3G (1)**	10.4	3	0	12	50	4.48
**Figure 3G (2)**	1	2	0	5	40	3.02
**Figure 3G (3)**	6	17	0	1	12	2.39
**Figure 3G (4)**	1.4	9	0	15	40	4

Supplementary table 2: Clinical information of RA patients for synovial tissue samples included in this study. C-reactive protein (CRP), erythrocyte sedimentation rate (ESR), swollen 28-joint count (SJC28), tender 28-joint count (TJC28) and visual analogue scale (VAS) at time of arthroscopy are shown.

Figure and sample	CRP	ESR	SJC28	TJC28	VAS	DAS28
**RNAseq sample 1**	7	30	14	14	-	-
**RNAseq sample 2**	9	17	5	3	75	4.43
**RNAseq sample 3**	4	13	0	0	40	2.1
**RNAseq sample 4**	2.5	27	6	6	30	3.89
**RNAseq sample 5**	25	79	4	2	50	4.18

Supplementary table 3: Clinical information of RA patients, donors of B cells used in RNAseq analysis. C-reactive protein (CRP), erythrocyte sedimentation rate (ESR), swollen 28-joint count (SJC28), tender 28-joint count (TJC28) and visual analogue scale (VAS) at time of arthroscopy are shown.

Supplementary Methods:

Differential Expression Analysis.

TPM count values obtained by pseudo alignment and quantification of raw read files (fastq's) with Kallisto were read into R using the *tximport* Bioconductor package, and transcript count values were summarized to gene level counts using the summarizeToGene command. The counts were then normalized for library size using edgeR's calcNormFactors function. Genes with low expression across samples were filtered out. Differential analysis was performed within edgeR by using edgeR's generalized linear model implementation (as embodied by the commands glmQLFit and glmQLFTest). In order to account for the pairwise origin of samples (i.e PD1+ and PD1- B cells were obtained in pairs from donors), and both a paired analysis accounting for the paired structure of the dtaa and an unpaired analysis was performed. The paired analysis yielded approximately 1100 differentially expressed genes out of a total of approximately 16600 genes that were present after filtering. The unpaired analysis reported a smaller group of genes (~900) that were differentially expressed. Downstream pathway analysis was performed using the results from the paired analysis. A volcano plot of the differentially expressed genes is shown in Fig 6.

Pathway Analysis.

Pathway analysis was performed by processing the list of differentially expressed genes using the Ingenuity Pathway Analysis (IPA) software from Qiagen. Only genes that differentially expressed with an FDR value of < 0.05 were used to perform the pathway analysis. In addition

to this, the differentially expressed genes that satisfied this FDR threshold were ranked by their significance and gene set enrichment analysis performed on them, using gene sets from the Broad Institute's MSigDb database.

Multiple pathway databases were used to test for enrichment, among them, were the database of GO terms as represented by MSigDb's c5.all.v.7.1, the canonical pathways database as represented by c2.cp.all.v.7.1 and the hallmarks of cancer database (h.all.v.7.1). There was common enrichment in gene sets or pathways related to the cell cycle (IPA: EIF2 signaling, $p = 3x10^{-58}$, KEGG: Cell Cycle with NES=1.77, FDR = 0.002, Hallmarks: G2M Checkpoint, NES=1.96, FDR = 0.004 & E2F Targets, NES=1.82, FDR = 0.004). Furthermore, there was common enrichment in the Glycolysis and Oxidative phosphorylation pathways as stated in the main text.

To understand heterogeneity in signaling between samples, single sample pathway enrichment analysis was performed using the GSVA (gene set variation analysis) method as implemented in the R/Bioconductor package of the same name, using the same gene set databases as used for GSEA. To illustrate these results, a heatmap of the GSVA enrichment is shown for the Hallmark gene set. We also show in the table below, results of the GSEA analysis of the differentially expressed genes.

NAME	SIZE	NES	NOM p-val	FDR q-val	FWER p-val	RANK AT MAX
HALLMARK_G2M_CHECKPOINT	27	1.9724258	0	0	0	127
HALLMARK_INFLAMMATORY_RESPONSE	22	1.7869042	0	0.01079322	0.019	107
HALLMARK_E2F_TARGETS	30	1.777163	0.00105042	0.00862043	0.023	86
HALLMARK_ESTROGEN_RESPONSE_LATE	21	1.7480316	0.00442478	0.00835823	0.03	131
HALLMARK_ALLOGRAFT_REJECTION	27	1.5741361	0.00853789	0.07547446	0.286	163

HALLMARK_TNFA_SIGNALING_VIA_NFKB	27	1.5510603	0.02136752	0.07770276	0.342	190
HALLMARK_APOPTOSIS	18	1.4774623	0.06081081	0.12813708	0.573	143
HALLMARK_IL2_STAT5_SIGNALING	31	1.380032	0.09957627	0.24689725	0.856	278
HALLMARK_GLYCOLYSIS	26	1.3773723	0.1025641	0.22290102	0.859	373
HALLMARK_COMPLEMENT	16	1.313388	0.16836159	0.2951678	0.955	298
